# Dynamics of an optomechanical system with quadratic coupling: Effect of first order correction to adiabatic elimination

**DOI:** 10.1038/srep35583

**Published:** 2016-10-18

**Authors:** Cheng Jiang, Yuanshun Cui, Guibin Chen

**Affiliations:** 1 School of Physics and Electronic Electrical Engineering, Huaiyin Normal University, 111 West Chang Jiang Road, Huaian 223300, China; 2 Jiangsu Key Laboratory of Modern Measurement Technology and Intelligent Systems, Huaiyin Normal University, 111 West Chang Jiang Road, Huaian 223300, China

## Abstract

We explore theoretically the dynamics of an optomechanical system in which a resonantly driven cavity mode is quadratically coupled to the displacement of a mechanical resonator. Considering the first order correction to adiabatic elimination, we obtain the analytical expression of optomechanical damping rate which is negative and depends on the position of the mechanical resonator. After comparing the numerical results between the full simulation of Langevin equations, adiabatic elimination, and first order correction to adiabatic elimination, we explain the dynamics of the system in terms of overall mechanical potential and optomechanical damping rate. The antidamping induced by radiation pressure can result in self-sustained oscillation of the mechanical resonator. Finally, we discuss the time evolution of the intracavity photon number, which also shows that the effect of first order correction cannot be neglected when the ratio of the cavity decay rate to the mechanical resonance frequency becomes smaller than a critical value.

The field of cavity optomechanics is concerned with the coupling between the cavity modes and mechanical degrees of freedom via radiation pressure, which has witnessed remarkable progress in the past decade (see refs 1, 2, 3 for recent reviews). The typical optomechanical system consists of a Fabry-Pérot cavity where one of the end mirrors can move. When the cavity is driven by a pump laser, the circulating light inside the cavity gives rise to radiation pressure force that deflects the mirror. Any displacement of the mirror will in turn change the cavity’s length and thus modulate the resonance frequency of the cavity. This dynamical back-action of photons caused by radiation pressure can influence the dynamics of the system, which depends on the frequency and power of the pump laser applied to the cavity. Applying a pump laser of a frequency red detued with respect to the cavity resonance allows for ground state cooling of the mechanical resonators^4^,^5^,^6^,^7^, quantum state transfer^8^,^9^,^10^, optomechanically induced transparency (OMIT)^11^,^12^,^13^,^14^,^15^,^16^, and light storage^17^. Recent efforts have shown that a blue-detuned pump laser can lead to self-sustained oscillations^18^,^19^,^20^,^21^,^22^,^23^ and even chaos^24^,^25^. More recently, attractor diagrams related with self-sustained oscillations have been experimentally demonstrated^26^,^27^,^28^. In addition, the pump laser is usually chosen to be resonant with the cavity field for the purpose of position, force, or mass sensing^29^,^30^,^31^. The above-mentioned achievements, as well as many others, have mainly relied on the optomechanical coupling that varies linearly with the displacement of the mechanical resonator.

However, some other optomechanical systems with quadratic coupling, i.e., the optical cavity mode is quadratically coupled to the displacement of the mechanical resonator, have also attracted extensive attention in recent years. Experimental realizations with this kind of optomechanical coupling include “membrane-in-the-middle” configuration where the membrane is placed at a node or antinode of the cavity mode^32^,^33^, or trapping either a cloud of ultracold atoms^34^ or levitating nanoparticles^35^ in an optical cavity. Such a quadratic interaction offers new possibilities to investigate cooling^36^, squeezing^37^,^38^, photon blockade^39^, and slow light^40^ in optomechanical systems. Specifically, Buchmann *et al*. investigated macroscopic tunneling of a membrane in an optomechanical double-well potential^41^. Seok *et al*. first studied theoretically the dynamics of multiple mechanical oscillators coupled to a single cavity field mode via quadratic optomechanical interactions^42^ and then they proposed a scheme for realizing dynamic stabilization of an optomechanical oscillator by modulating the input power^43^. In refs 41, 42, 43, the authors mainly consider the situation where the cavity decay rate *κ* is much larger than the resonance frequency *ω*_*m*_ of the mechanical oscillator, therefore adiabatic elimination of the cavity field is valid. However, when the above condition is not satisfied, some correction to adiabatic elimination is needed to show the correct dynamics of the system. In the present paper, we first compare the numerical results between full simulation of Langevin equations, adiabatic elimination, and first order correction to adiabatic elimination by varying the value of *κ*/*ω*_*m*_. It is seen that the effect of first order correction to adiabatic elimination becomes more important when *κ*/*ω*_*m*_ reduces. After considering the first order term of *ω*_*m*_/*κ*, we find that overall mechanical potential keeps the same with adiabatic elimination but there is an additional optomechanical damping rate which is negative in the chosen parameter regimes. The antidamping rate becomes larger when *κ*/*ω*_*m*_ reduces, which can finally lead to self-sustained oscillation of the mechanical resonator. Therefore, the model in this paper can successfully predict interesting dynamics such as self-sustained oscillation only after including the first order correction to adiabatic elimination. Such nonlinear dynamics are drawing considerable interest in cavity optomechanics but have seldom been investigated with only quadratic optomechanical coupling. On the other hand, quadratic coupling in principle makes the system less susceptible to chaotic dynamics and thus may provide a new way of studying nonlinear dynamics in cavity optomechanical systems.

## Results

### Model and Methods

We consider an optomechanical system in which a driven cavity of resonance frequency *ω*_*c*_ is quadratically coupled to the displacement of a mechanical resonator of effective mass *m* and frequency *ω*_*m*_. The Hamiltonian of the system can be written as


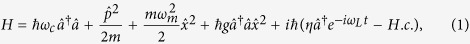


where the first term describes the energy of the cavity field with 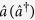 being the annihilation (creation) operator such that 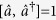. The next two terms represent the energy of the free mechanical resonator, where 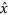 and 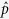 are the position and momentum operators for the mechanics with the commutation relation 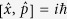. The fourth term describes the quadratic optomechanical interaction where *g* is the quadratic optomechanical coupling constant. In the “membrane-in-the-middle” optomechanical system, 
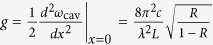
44, where *λ* is the wavelength of the pump field, *L* is the length of the cavity, *R* is the reflectivity of the membrane, and *c* is the speed of light in a vacuum. This dispersive quadratic coupling *g*/2*π* can now reach 30 MHz/nm^2^ for a thin dielectric membrane^33^. The single-photon quadratic coupling strength is then given by 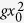, where 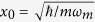 is the zero-point fluctuation of the mechanical resonator^37^. It is assumed throughout this paper that *g* is negative-valued as is appropriate to trapping around a maximum of the cavity intensity^42^,^43^. Finally, the last term represents the coupling between the cavity field and the pump field of frequency *ω*_*L*_ with a rate 
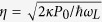
, where *κ* is the cavity decay rate and *P*_0_ is the input power of the pump field. In the rotating frame of the pump frequency *ω*_*L*_, the classical equations of motion can be derived by replacing operators by their c-number equivalent in the Heisenberg-Langevin equations:


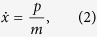



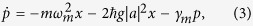



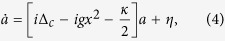


where Δ_*c*_ = *ω*_*L*_ − *ω*_*c*_ is the cavity detuning and *γ*_*m*_ is the mechanical damping rate. According to Eq. (4), we can obtain


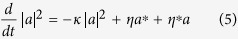


Defining the intensity of the cavity field *I* = a^*^a = |*a*|^2^, normalized time *τ* = *ω*_*m*_*t*^19^, we can easily get the following equations according to Eqs (4) and (5):


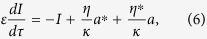



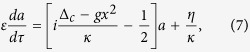


where we have introduced a small dimensionless parameter 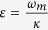 in order to expand the quantities *a* and *I*. Given stable parameters, the cavity field approaches its steady state in a time scale *κ*^−1^. When *κ* ≫ *ω*_*m*_, thus *ε* is a small parameter, the cavity field adiabatically follows the position of the mechanical resonator, and the dynamics of the resonator are robust against the fluctuations of the cavity field (adiabatic elimination is valid). Therefore, the cavity field stays in its quasi-steady state (*da*/*dτ* = 0) and can be given by


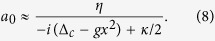


Adiabatic elimination of the cavity field in optomechanical systems with quadratic coupling has been widely used in previous works, see refs 41, 42, 43 for example. Substituting Eq. (8) into Eq. (3) then gives the temporal evolution of the momentum of the mechanical resonator^42^,^43^. However, if *ε* becomes larger, we have to consider higher order terms of *ε*. The cavity field *a* and *I* can be expanded in powers of *ε*, i.e., 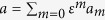 and 
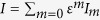
43. One can in principle solve the differential Eqs (6) and (7) to arbitrary order. In this paper, we mainly consider the regime where first order correction to adiabatic elimination is dominant. So we can expand *I* and *a* in powers of *ε* to the first order, i.e.,


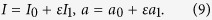


Upon substituting Eq. (9) into Eqs (6) and (7) and working to the first order of *ε*, one can have the following coupled equations by comparing the coefficients of *ε*^0^ and *ε*^1^
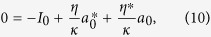

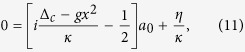

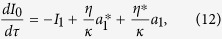

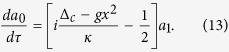
We can see that Eqs (10) and (11) can give the results of adiabatic elimination and Eqs (12) and (13) correspond to the first order terms of *ε*. Given that *I* is equal to |*a*|^2^, it is easy to check that *I*_0_ = |*a*_0_|^2^ and 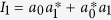 according to Eqs (10)–(13), 23, 24, 25, and we can obtain
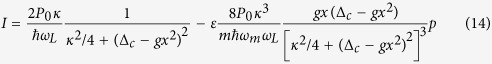
The first term in the right side of Eq. (14) is the result of adiabatic elimination^43^, and the second term represents the first order correction. Substituting Eq. (14) into Eq. (3) then gives
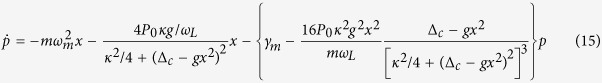
From this equation we can see that besides the intrinsic mechanical damping rate *γ*_*m*_, there is an additional damping term^45^, which can be named as optomechanically induced damping rate and is given by
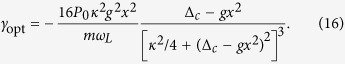
If *γ*_opt_ = 0, Eq. (15) is changed to the Newton’s equation of motion for the mechanics with adiabatic elimination.

In the absence of mechanical dissipation, Eq. (15) for the mechanical system can be put in the canonical form 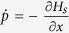, with the Hamiltonian
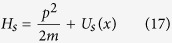
and overall mechanical potential
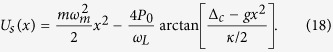
The slowly changing potential is the sum of the harmonic potential and arctangent function which can be modified by the pump field, quadratic coupling constant *g*, and cavity decay rate *κ*. In this paper, we are mainly interested in the case where *g* < 0. When the pump power is greater than a critical value, the overall potential exhibits a symmetric double-well potential centered on *x* = 0^41^,^43^.

### Dynamics of the system

**Figure 1:**
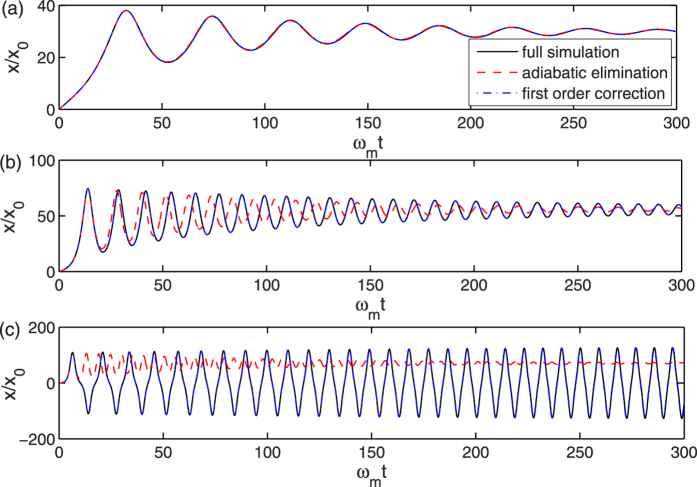
Numerical results of the time evolution of the normalized position *x*/*x*_0_ based on full simulation, adiabatic elimination, and first order correction when *κ*/*ω*_*m*_ is equal to (**a**) 200, (**b**) 180 and (**c**) 100, respectively. The other parameters are 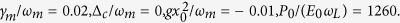

The dynamics of the optomechanical system can be investigated by directly solving the classical Eqs (2)–(4), 12, 13. However, in order to obtain more physical understandings, we first compare the numerical results between full simulation of Eqs (2)–(4), 12, 13, adiabatic elimination, and first order correction to adiabatic elimination of Eq. (2) and Eq. (14). In our simulations, the position, momentum, and energy of the mechanical mode are measured in units of the natural length 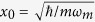, momentum 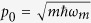, and energy *E*_0_ = *ħω*_*m*_^43^, respectively. Moreover, all the frequencies (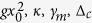) are scaled in units of *ω*_*m*_^19^. The Runge-Kutta method is used to solve these ordinary differential equations, and we use *a*|_*t*=0_ = 0, *x*|_*t*=0_ = 0, and *p*/*p*_0_|_*t*=0_ = 0.7126 as the initial condition^43^, which can be achieved by cooling the mechanical resonator to the ground state of motion^4^,^5^,^6^,^7^. As shown in Fig. 1, the normalized position *x*/*x*_0_ is plotted as a function of *ω*_*m*_*t* when *κ*/*ω*_*m*_ = 200, 180, and 100, respectively. In the present paper, we mainly consider the situation where the pump field is resonant with the cavity field, i.e., Δ_*c*_ = 0. We can see from Fig. 1(a) that when *κ*/*ω*_*m*_ = 200, 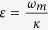 is a small parameter, higher order terms of *ε* can be neglected and adiabatic elimination is valid, therefore, three numerical results coincide with each other. However, if *κ*/*ω*_*m*_ is reduced to 180, *ε* becomes larger, the effect of first order term of *ε* cannot be neglected. As shown in Fig. 1(b), the result of adiabatic elimination (red dashed line) is a little different from that of full simulation (black solid line), but the first order correction (blue dash dotted line) still agrees well. If *κ*/*ω*_*m*_ is further reduced to 100, it can be seen clearly that after a transient process the result of adiabatic elimination is quite different from those of full simulation and first order correction. If we adiabatically eliminate the cavity field, the mechanical oscillator should finally decay to a fixed position. But the full simulation of Eqs (2)–(4), 12, 13 shows that self-sustained oscillation appears when *κ*/*ω*_*m*_ reaches a critical value. The smaller value of *κ*/*ω*_*m*_, the worse of adiabatic elimination is. However, we have checked that as long as *κ*/*ω*_*m*_ is bigger than 50, first order correction to adiabatic elimination is still valid. In addition, Fig. 1 indicates that when the value of *κ*/*ω*_*m*_ is changed, the oscillation frequency and the final equilibrium position of the mechanical resonator is different, which can be explained by the overall mechanical potential and effective mechanical damping rate.

**Figure 2:**
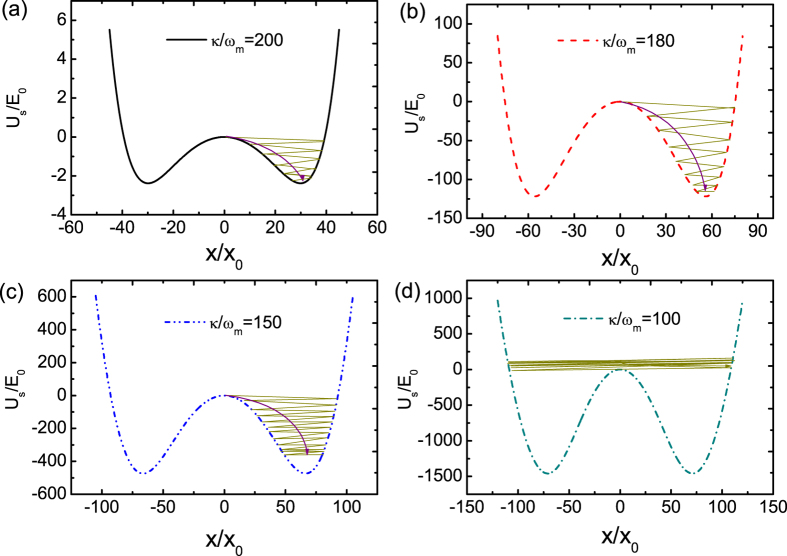
Overall mechanical potential for four different values of *κ*/*ω*_*m*_. (**a**) *κ*/*ω*_*m*_ = 200, (**b**) *κ*/*ω*_*m*_ = 180, (**c**) *κ*/*ω*_*m*_ = 150, and (**d**) *κ*/*ω*_*m*_ = 100. Other parameters are the same as in Fig. 1.

Figure 2 plots the overall mechanical potential *U*_*s*_/*E*_0_ as a function of the normalized position *x*/*x*_0_ when *κ*/*ω*_*m*_ equals to 200, 180, 150, and 100, respectively, which shows a double-well structure. In the center *x*/*x*_0_ = 0, there is a local maximum value, therefore the mechanical oscillator is unstable at this position. If the mechanical resonator starts to oscillate in the center of the double-well, it will eventually relax into either the bottom of the double-well due to mechanical damping, as shown in Fig. 2(a–c). However, when *κ*/*ω*_*m*_ changes, the width and depth of the double-well potential also varies. The position of the minimum value of the double-well potential can be obtained when the first derivative of the potential equals to zero. If we define the normalized potential


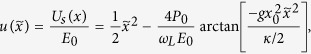


then one can get 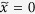 or


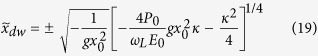


by setting 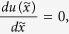 where 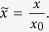 Substituting the parameters into Eq. (19), we can find that the analytical results are consistent with Fig. 1(a,b).

**Figure 3:**
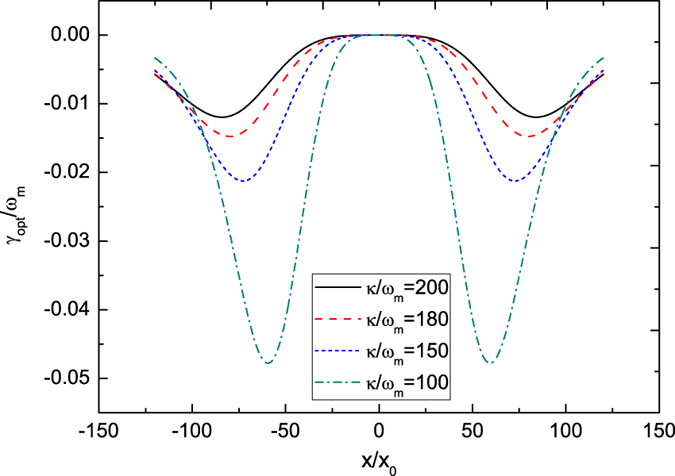
Normalized optomechanical damping rate *γ*_opt_/*ω*_*m*_ versus normalized position *x*/*x*_0_ when *κ*/*ω*_*m*_ equals to 200, 180, 150, and 100, respectively. Other parameters are the same as in Fig. 1.

On the other hand, Figs 1(c) and 2(d) indicate that the amplitude of the mechanical oscillation will keep almost the same when *κ*/*ω*_*m*_ = 100, which can be called self-sustained oscillations. The reason for this phenomenon can be explained in terms of effective mechanical damping rate. The effective mechanical damping rate is the sum of intrinsic and optomechanical damping rates, i.e.,


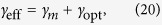


where *γ*_opt_ is given by Eq. (16). Figure 3 plots the normalized optomechanical damping rate *γ*_opt_/*ω*_*m*_ versus normalized position *x*/*x*_0_ when *κ*/*ω*_*m*_ = 200, 180, 150, and 100, respectively. It should be noted that here Δ_*c*_ = 0 and 
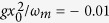
. We can see that optomechanical damping rate is negative and depends on the position of the mechanical resonator. Generally, when *κ*/*ω*_*m*_ reduces, the antidamping becomes larger, which can lead to amplification of thermal fluctuations and finally to an instability if the full damping rates becomes negative *γ*_eff_ < 0. In our simulations, we have chosen *γ*_*m*_/*ω*_*m*_ = 0.02. When *κ*/*ω*_*m*_ = 200 and 180, the antidamping is smaller than 0.02, therefore the effective damping rate is still positive and mechanical resonator should decay to either the bottom of the double-well potential, as shown in Fig. 1(a,b). When *κ*/*ω*_*m*_ = 100, there are two regions where antidamping is larger than the intrinsic mechanical damping rate but antidamping is smaller in other regions. At the beginning, the negative *γ*_opt_ decreases the effective damping rate and leads to heating of the mechanical resonator. Once overall damping rate *γ*_*m*_ + *γ*_opt_ becomes negative, an instability ensues and mechanical oscillation will at first grow exponentially in time. Later, the nonlinearity of the optomechanical coupling will saturate the growth of the mechanical oscillation amplitude, resulting in a steady-state regime with oscillations at a fixed amplitude, as shown in Fig. 1(c). When the mechanical resonator is driven in large amplitude, mechanical nonlinearity such as the Duffing effect should be considered. Discussions about the effect of the mechanical nonlinearity in self-sustained oscillation can be found in refs 20 and 22. In this paper we didn’t consider the mechanical nonlinearity.

Furthermore, the approximate expression of the oscillation frequency in the double-well can be derived by doing Taylor expansion to the second order at the position 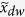. Supposing that


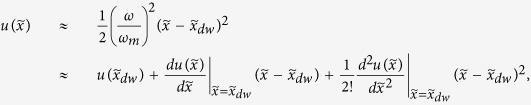


the oscillation frequency around the bottom of the double-well is then given by


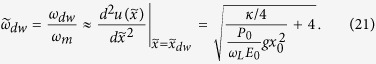


**Figure 4:**
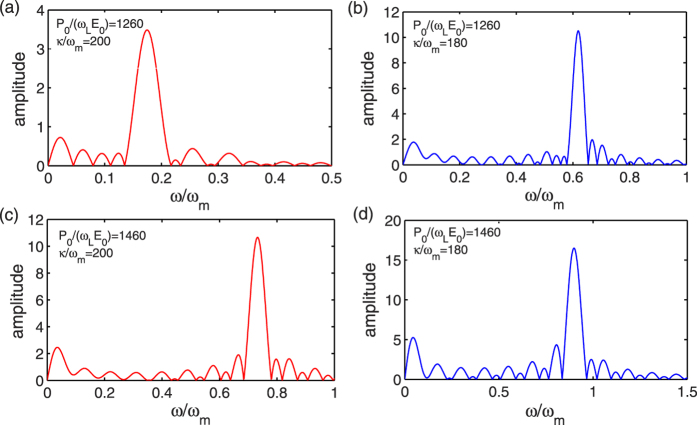
Frequency spectra of the mechanical oscillator under different pumping amplitudes and cavity decay rates. (**a**) *P*_0_/(*ω*_*L*_*E*_0_) = 1260 and *κ*/*ω*_*m*_ = 200, (**b**) *P*_0_/(*ω*_*L*_*E*_0_) = 1260 and *κ*/*ω*_*m*_ = 180, (**c**) *P*_0_/(*ω*_*L*_*E*_0_) = 1460 and *κ*/*ω*_*m*_ = 200, and (**d**) *P*_0_/(*ω*_*L*_*E*_0_) = 1460 and *κ*/*ω*_*m*_ = 180. Other parameters are the same as in Fig. 1.

Figure 1 also shows that the oscillation period almost keeps the same after a transient process, with the damping rate given by the effective mechanical damping rate. The oscillation frequency can be approximately expressed by Eq. (21). To verify this, we do the fast Fourier transform after the transient process. Figure 4 plots the frequency spectra of the mechanical resonator under different pumping amplitudes and cavity decay rates. It can be seen that when *P*_0_/(*ω*_*L*_*E*_0_) or *κ*/*ω*_*m*_ is changed, the position of the resonance peak varies. Substituting the specific parameters into Eq. (21), we can find that the analytical results are nearly consistent with the numerical results. Therefore, under the action of radiation pressure force, when the mechanical resonator relaxes into either well of the double-well potential, it undergoes the damped vibration, with normalized oscillation frequency approximately given by Eq. (21).

**Figure 5:**
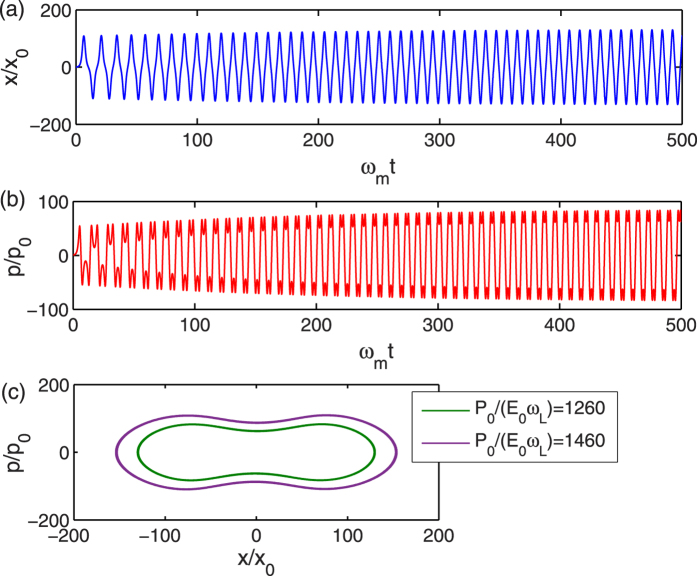
Normalized position *x*/*x*_0_ (**a**) and momentum *p*/*p*_0_ (**b**) as a function of *ω*_*m*_*t* when *κ*/*ω*_*m*_ = 100. (**c**) Phase-space trajectories of the mechanical oscillator when *P*_0_/(*ω*_*L*_*E*_0_) = 1260 and 1460, respectively. Other parameters are the same as in Fig. 1.

In the following, we will investigate the self-sustained oscillation in this optomechanical system in more details. From the numerical solutions of Eqs (2) and (15), we plot the time evolution of the normalized position *x*/*x*_0_, momentum *p*/*p*_0_, and the corresponding limit cycle in the phase space of the mechanical resonator in Fig. 5. As discussed above, when *κ*/*ω*_*m*_ = 100, the effective mechanical damping rate is negative in some regions, and the amplitudes of position and momentum will be fixed in the long-time limit due to the nonlinearity inherent to the optomechanical coupling, which can be clearly seen in Fig. 5(a,b). Therefore, the trajectory of mechanical motion in phase space can constitute a closed loop or limit cycle, as shown in Fig. 5(c). Each limit cycle corresponds to a stable self-sustained oscillation in the long-time limit, which should satisfy the energy-balance condition, i.e., the energy dissipated from friction must be equal to the energy gained from optical radiation pressure force in one whole cycle. When the pump power increases, the mechanical resonator gains more energy from optical radiation pressure, therefore, the amplitudes of the mechanical motion should also become bigger. This can be clearly seen in Fig. 5(c), where the outer and inner loop corresponds to *P*_0_/(*ω*_*L*_*E*_0_) = 1460 and 1260, respectively. Based on the energy-balance condition, several kinds of attractor diagrams have been investigated both theoretically^21^,^22^ and experimentally^26^,^27^,^28^ in optomechanical systems.

**Figure 6:**
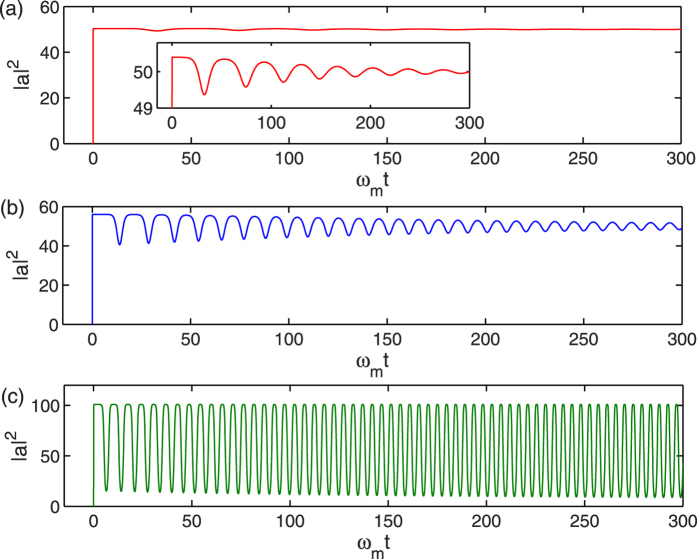
Photon number of the cavity field |*a*|^2^ as a function of *ω*_*m*_*t* when *κ*/*ω*_*m*_ is equal to (**a**) 200, (**b**) 180, and (**c**) 100, respectively. The inset of Fig. 6(a) is the enlargement of the photon number between 49 and 51, which shows the small change of the photon number. Other parameters are the same as in Fig. 1.

In the end, the effect of first order correction to adiabatic elimination is studied by considering the numerical results of intracavity photon number |*a*|^2^. Figure 6 shows the time evolution of photon number of the cavity field |*a*|^2^ for *κ*/*ω*_*m*_ = 200, 180, and 100, respectively. When *κ*/*ω*_*m*_ is equal to 200, it can be seen from Fig. 6(a) that the photon number suddenly jumps from zero to a large number and then almost keeps constant as time passes. Therefore, adiabatic elimination is valid in this case and the numerical results of adiabatic elimination, first order correction, and full simulation coincide with each other, as shown in Fig. 1(a). However, the inset of Fig. 6(a) shows that there is actually a small variation of photon number |*a*|^2^. If *κ*/*ω*_*m*_ is reduced to 180, we can see from Fig. 6(b) that the variation of photon number is evident and the average photon number is bigger. In this case, the first order term in Eq. (9) can no longer be neglected and adiabatic elimination cannot show the correct dynamics of the system, which can be seen from Fig. 1(b). If *κ*/*ω*_*m*_ is further reduced to 100, the photon number undergoes nearly periodic oscillation with big amplitude after a transient process and therefore adiabatic elimination is completely invalid. However, first order correction to adiabatic elimination can still show almost the same numerical results with the full simulation of Eqs (2)–(4), 12, 13. Based on the above discussions, we can see that when the optomechanical system works in the unresolved sideband regime, if *κ*/*ω*_*m*_ is big enough, the dynamics of the system can be analyzed by employing adiabatic elimination of the cavity field. But if the value of *κ*/*ω*_*m*_ is reduced to a certain range, first order correction to adiabatic elimination is good enough. However, when *κ*/*ω*_*m*_ is much smaller, other higher order terms of *ω*_*m*_/*κ* have to be considered. It should be pointed out that the parameters we choose in this paper are the same as in the ref. 43, where the ratio of the quadratic coupling strength 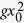 to the mechanical resonance frequency *ω*_*m*_ is −0.01. In the recent experiment using a tunable photonic crystal optomechanical cavity^46^, this value is about 10^−4^. In addition, it has been proposed that quadratic optomechanical coupling can be simulated in a superconducting electrical circuit^47^, where the quadratic coupling strength can be greatly improved and even reach the strong-coupling regime. If we choose 
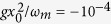
, much higher pump power *P*_0_ will be needed to get negative enough optomechanical damping rate and generate the double-well potential, as can be seen from Eqs (16) and (18). However, we have checked numerically that our results obtained above are still valid.

## Discussion

In summary, we have investigated the dynamics of a quadratically coupled optomechanical system in the unresolved sideband regime. When the optical cavity is driven by a resonant laser field and the quadratic optomechanical coupling constant is negative, we obtained a negative and position-dependent optomechanical damping rate after considering the first order correction to adiabatic elimination. By comparing the numerical results between full simulation of Langevin equations, adiabatic elimination, and first order correction, we found that adiabatic elimination is no longer valid when the cavity decay rate is smaller than a certain value, therefore first order correction is needed to show the correct dynamics of the system. The physical reason for the dynamics can be well understood by the double-well potential and optomechanical damping rate. We also discussed self-sustained oscillation of the mechanical resonator and time evolution of the intracavity photon number in this optomechanical system. Therefore, our work provided a simple and analytical method to investigate the dynamics of optomechanical systems with quadratic coupling.

## Additional Information

**How to cite this article**: Jiang, C. *et al*. Dynamics of an optomechanical system with quadratic coupling: Effect of first order correction to adiabatic elimination. *Sci. Rep.* **6**, 35583; doi: 10.1038/srep35583 (2016).
